# Data on seed priming and seedling growth of Barli 21 tobacco varieties under polyethylene glycol and salinity stress conditions

**DOI:** 10.1016/j.dib.2018.08.033

**Published:** 2018-08-16

**Authors:** Fatemeh Nejatzadeh-Barandozi

**Affiliations:** Department of Horticulture, Faculty of Agriculture, Khoy Branch, Islamic Azad University, Khoy, Iran

**Keywords:** Polyethylene glycol (PEG), Tobacco, Salinity, Emergence percentage

## Abstract

Data on the effect of seed priming on Barli 21 tobacco (*Nicotiana tobacum* L.) cultivars, an experiment was carried out in 2014 at the Tobacco Research center of Urmia, Iran under saline and Polyethylene glycol conditions. This experiment was arranged as factorial, based on RCB design with three replications. Treatments were polyethylene glycol (− 0.5%, − 1%, − 1.5% and − 2% (PEG) and hydropriming, and salinity levels (1, 2, 3 and 4 dS m^−1^ KNO_3_) in periods 1, 2, 3, 5 and 10 days. Means of Emergence time, emergence rate coefficient, Emergence rate index, Emergence rate, and Emergence percentage decreased with increasing salinity. Emergence time and emergence rate coefficient increased with hydropriming in priming 5 and 10 days. Emergence rate index, Emergence rate, and Emergence percentage increased with 1.5% Polyethylene glycol. Seed priming with Polyethylene glycol was more beneficial in improving Emergence percentage, compared with KNO_3_ priming.

**Specifications Table**

**Table:** 

Subject area	*chemistry, biology*
More specific subject area	Seed Priming and Seedling Growth of Barli 21 Tobacco data Under PEG and Salinity stress
Type of data	*Table and figure*
How data was acquired	*Laboratory experiments*
Data format	*Analyzed data*
Experimental factors	*Seed Priming and Seedling Growth of Barli 21 Tobacco, PEG, Salinity stress*
Experimental features	*Seed treatments were polyethylene glycol (*− *0.5*%*,* − *1*%*,* − *1.5*% *and* − *2% (PEG) and hydropriming, and salinity levels (1, 2, 3 and 4 dS m^−1^ KNO3) in periods 1, 2, 3, 5 and 10 days. seedling emergence was counted daily with seeds recorded as emerged, when hypocotyls appeared and mean emergence rate was calculated according to Ellis and Roberts (1980). After emergence, Emergence time, emergence rate coefficient, Emergence rate index, Emergence rate, and Emergence percentage were determined*
Data source location	*Urmia, Iran*
Data accessibility	*All data are present in this article*

**Value of the data**

• These data provide the priming of seed and seedling growth of Barli 21 Tobacco under PEG (*polyethylene glycol)* and salinity stress and relationships between seed priming and salinity stress it shows.
• PEG (*polyethylene glycol*) treatment may be applied to improving germination of Tobacco seeds as valuable method for increasing hardening at salinity stress conditions.
• These data are valuable to researchers investigating priming technique to improving germination of Barli 21 Tobacco seed at salinity stress.

## 1 Data

**Fig. 1:**
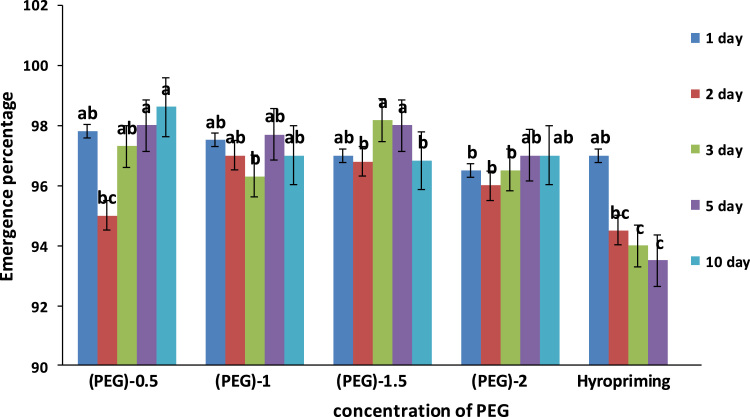
Means of interaction of priming duration × concentration of polyethylene glycol and hydropriming on emergence percentage of tobacco. Different letters in each column indicating significant difference at *p* ≤ 0.05.

**Fig. 2:**
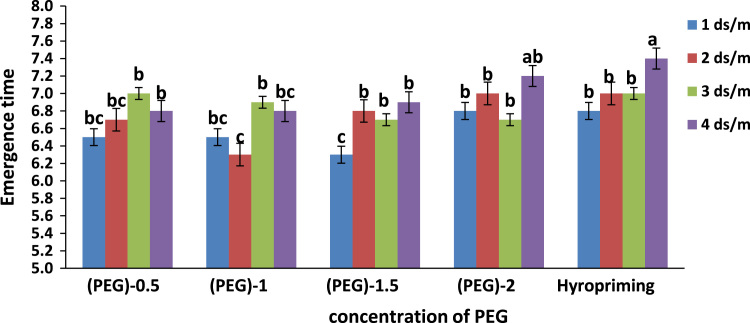
Means of interaction of salinity × concentration of polyethylene glycol and hydropriming on Emergence time of tobacco. Different letters in each column indicating significant difference at *p* ≤ 0.05.

**Fig. 3:**
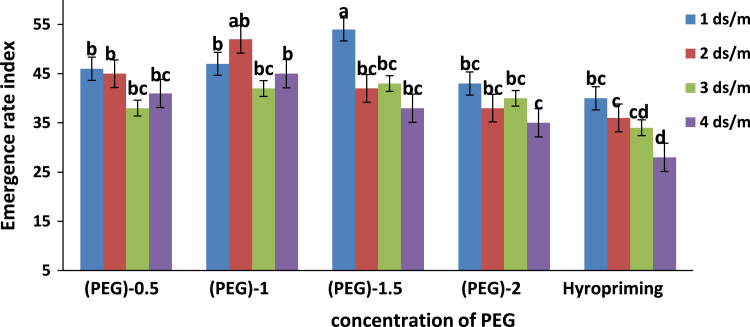
Means of interaction of salinity × concentration of polyethylene glycol and hyropriming on Emergence rate index of tobacco. Different letters in each column indicating significant difference at *p* ≤ 0.05.

Effect of Polyethylene glycol and hydro priming treatments on Emergence traits of tobacco seed are presented in Table 1. Effect of priming time on emergence traits of tobacco are presented in comparison table (Table 2). Means of emergence traits of tobacco affected by salinity treatments are presented in Table 3. In addition, Analyses of variance of the effects of priming, priming time on emergence traits of tobacco under salinity stress are presented in Table 4. Means of interaction of priming duration × concentration of polyethylene glycol and hydropriming on emergence percentage of tobacco are presented in Fig. 1. Also Means of interaction of salinity ×concentration of polyethylene glycol and hydropriming on Emergence time of tobacco are presented in Fig. 2. In addition, means of interaction of salinity × concentration of polyethylene glycol and hyropriming on Emergence rate index of tobacco are presented in Fig. 3.

**Table 1:** Means of emergence traits of tobacco affected by Polyethylene glycol and hydro priming treatments.

Treatment	Emergence time (day)	Emergence rate coefficient (%)	Emergence rate index (seed day^−^^1^)	Emergence rate (% day^−^^1^)	Emergence percentage (%)
PEG-0.5	6.70 bc	2.1 b	42 ab	42 ab	97 a
PEG-1	6.52 c	2.2 b	46 a	45 a	96 a
PEG-1.5	6.55 c	2.1 b	45 a	44 a	98 a
PEG-2	6.90 ab	2.2 b	38 bc	40 b	96 a
Hydropriming	7.00 a	2.5 a	35 c	35 c	91 b

Different letters in each column indicating significant difference at *p* ≤ 0.05.

**Table 2:** Means of emergence traits of tobacco affected by priming duration.

Treatment	Emergence time (day)	Emergence rate coefficient (%)	Emergence rate index (seed day^−^^1^)	Emergence rate (% day^−1^)	Emergence Percentage (%)
Priming 1 day	6.6 b	2.1 b	1.9 b	44 a	97 a
Priming 2 day	6.8 ab	2.2 b	2.0 ab	39 ab	96 ab
Priming 3 day	6.78 ab	2.1 b	2.02 ab	41 ab	95 ab
Priming 5 day	6.9 a	2.2 b	2.01 ab	37 b	96 ab
Priming 10 day	6.77 ab	2.5 a	2.1 a	40 ab	94 b

Different letters in each column indicating significant difference at *p* ≤ 0.05.

**Table 3:** Means of emergence traits of tobacco affected by salinity treatments.

Salinity treatment	Emergence time (day)	Emergence rate index (seed day^−1^)	Emergence rate (% day^−1^)
1 ds/m	6.4 c	45 a	45 a
2 ds/m	6.5 bc	41 b	41 b
3 ds/m	6.8 ab	39 bc	39 bc
4 ds/m	7.0 a	35 c	37 c

Different letters in each column indicating significant difference at *p* ≤ 0.05.

**Table 4:** Analyses of variance of the effects of priming, priming time on emergence traits of tobacco under salinity stress.

Source of variation	Df	Emergence time (day)	Emergence rate coefficient (%)	Emergence rate index (seed day^−1^)	Emergence rate (% day^−^^1^)	Emergence percentage (%)
Priming (A)	4	2.021**	0.147**	1258.4**	848.9**	230.5 **
Priming time (B)	4	0.664*	0.027*	307.45 *	202.08**	36.76*
Salinity (C)	3	2.034**	0.0094**	1031.14**	636.6**	22.22 ns
(A × B)	16	0.278 ns	0.0308 ns	138.69 ns	90.42 ns	40.66**
(A × C)	12	0.356*	0.0042 ns	172.11*	105.17 ns	9.36 ns
(B × C)	12	0.0322 ns	0.0080 ns	12.74 ns	8.68 ns	12.50 ns
(A × B × C)	48	0.276 ns	0.0066 ns	132.63 ns	85.52 ns	10.08 ns
Error	198	0.195	0.008	93.29	58.62	5058.6
%CV		6.51	4.46	23.3	18.42	3.59

ns, not significant, * and **: significant at 1% and 5% respectively.

## 2 Experimental design, materials, and methods

An experiment was conducted to evaluate emergence traits and emergence percentage of tobacco under PEG and saline conditions in different periods. Treatments were polyethylene glycol (− 0.5%, − 1%, − 1.5% and − 2% (PEG)) and hydropriming, and salinity levels (1, 2, 3 and 4 dS m^−1^ KNO_3_) in periods 1, 2, 3, 5 and 10 days. Seeds of tobacco (*Nicotiana tobacum* L.) were divided into three subsamples, one of which was kept as control (hydropriming) and two other sub-samples were prepared for priming. A sub-sample was soaked in (PEG _6000_) solution and another one was pretreated with KNO_3_ solution with electrical conductivity of 12.5 dS m^−1^ at 15 °C for four hours [1], [2]. After priming, seeds were thoroughly washed with distilled water for a minute and then dried back to primary moisture at 20–23 °C in the laboratory. The greenhouse experiment was conducted at the Tobacco Research center of Urmia in 2014. This experiment was arranged as factorial, based on RCB (Randomized Complete Block) design with three replications. Ten seeds were sown 1 cm deep in each Petridis. Salinity treatments (1, 2, 3, 4 dS m^−1^) were applied immediately after sowing. Water and saline solutions were added to the Petridis. After emergence, seedling emergence was counted daily with seeds recorded as emerged, when hypocotyls appeared and mean emergence rate was calculated according to Ellis and Roberts (1980) [3]. After emergence, Emergence time, emergence rate coefficient, Emergence rate index, Emergence rate, and Emergence percentage were determined.

### 2.1 Germination assays

Response to priming was assessed by germination performance (rate, uniformity, total germination percentage, mean germination time and germination index). Germination was expressed as the cumulative percentage of germinated seeds. Mean germination time (*MGT*) was calculated according to the equation of Ellis and Roberts (1980) [3].

MGT=ΣDn/Σn

where *n* is the number of seeds, which were germinated on day *D* and *D* is the number of days counted from the beginning of germination.

Rate of germination (*R*) was calculated following modified formula [3]:

R=1/MGT

Uniformity (*GU*) was calculated following modified formula:

$$GU=\Sigma n/\Sigma \left[{\left(\Gamma n-t\right)}^{2}n\right]$$

where *t* is the time in days, starting from days 0, the day of germination and n is the number of seeds germinate *t* and Γ is equal to *MGT*.

Germination index (*GI*) was calculated as described in the Association of Official Seed Analysts (1983) as the following formulae:

*GI* = No. of germinated seeds/Days of first count+-------+ No. of germinated seeds/Days of final count

### 2.2 Statistical analysis

Analysis of variance of the data was carried out using MSTATC software. Duncan test was applied to compare means of each trait at *p* ≤ 0.05. EXCEL software was used to draw figures.
