# Supplementary material for: Data on seed priming and seedling growth of Barli 21 tobacco varieties under polyethylene glycol and salinity stress conditions
Source: Data Brief. 2018 Aug 16;20:454–8. doi: 10.1016/j.dib.2018.08.033 (PMC6122367; doi:10.1016/j.dib.2018.08.033)
Supplement: Supplementary file 1 — Transparency document [file mmc1.doc]

# CONFLICT OF INTEREST FORM

Manuscript Title: *Data on* Seed Priming and Seedling Growth of Barli 21 Tobacco Varieties under Polyethylene Glycol and Salinity Stress Conditions

Ms Number: DIB-D-18-01185

Author(s): Fatemeh Nejatzadeh

**PLEASE NOTE:** As an integral part of the online submission process, each manuscript must contain a declaration of interests from ALL authors. Corresponding authors are required to confirm whether they and/or their co-authors have any competing interests to declare, and to provide details of these. The corresponding author is required to complete and upload this Declaration of Competing Interests form when submitting a manuscript to Data in brief via our online submissions system. It is the corresponding author’s responsibility to ensure that all authors adhere to this policy.

Data in brief requires disclosure by all authors of any financial interests or connections, direct or indirect, or other situations which may raise the question of bias in the work reported or the conclusions, implications, or opinions stated. **When considering whether you should declare a competing interest or connection please consider the competing interest test: Is there any arrangement that would embarrass you or any of your co-authors if it was to emerge after publication and you had not declared it?**

If the manuscript is published, this declaration of competing interest’s information may be communicated in a statement in the published paper. If an author states that there are no competing interests then a statement to this effect may be included in the published paper.

**Please complete either section I or section II**

**I. The corresponding author confirms on behalf of all authors that there have been no involvements that might raise the question of bias in the work reported or in the conclusions, implications, or opinions stated:**

Printed Name (corresponding author): Fatemeh Nejatzadeh

Signature: fnejatzadeh

Date: 30 June 2018
